# Two cases of Legionnaires’ disease due to *Legionella cardiaca*

**DOI:** 10.1128/asmcr.00043-24

**Published:** 2024-12-12

**Authors:** Xiang Y. Han, Christopher J. Bowman, Micah M. Bhatti, Diwaker Balachandran

**Affiliations:** 1 Department of Laboratory Medicine, The University of Texas MD Anderson Cancer Center, Houston, Texas, USA; 2 Department of Pulmonary Medicine, The University of Texas MD Anderson Cancer Center, Houston, Texas, USA; Vanderbilt University Medical Center, Nashville, Tennessee, USA

**Keywords:** Legionnaires’ disease, *Legionella*, *Legionella cardiaca*

## Abstract

**Background:**

*Legionella cardiaca* is a Gram-negative bacterium initially isolated from the heart valve of a patient with endocarditis. Since its species description in 2012, there have been no other report of infections.

**Case Summary:**

Here, we describe two cases of *L. cardiaca* pneumonia. The patients were a 74-year-old man from New Orleans, Louisiana, USA, with underlying multiple myeloma and a 67-year-old man from Oklahoma City, Oklahoma, USA, with chronic lymphocytic leukemia. They developed cough and fever while undergoing antineoplastic therapy in August 2022 and July 2024, respectively. Laboratory examinations showed leukocytosis in the man with multiple myeloma and pancytopenia in the other with leukemia. In both patients, chest computed tomography showed ground-glass and consolidative opacities in the lung fields to suggest pneumonia. The cultures of the bronchoalveolar lavage fluids grew slender Gram-negative bacilli on buffered charcoal yeast extract (BCYE) agar with growth dependence on L-cysteine. The bacilli were identified by sequencing analyses of the 16S rRNA gene and Mip gene as *L. cardiaca*. The patients were treated with a combination of cefepime and other antibiotics and recovered. Both cases were sporadic infections, with likely exposure in the patients’ hometowns of New Orleans and Oklahoma City, respectively.

**Conclusion:**

These results suggest that *L. cardiaca* is an opportunistic pathogen and may cause pneumonia, or Legionnaires’ disease, in immunocompromised patients. Culture with BCYE agar and gene sequencing analysis may be required to recover and identify the bacterium for an etiologic diagnosis.

## INTRODUCTION

Legionnaires’ disease is a type of pneumonia caused by *Legionella pneumophila* and other *Legionella* species (1, 2). The genus *Legionella* contains 66 or so species of Gram-negative environmental bacteria and several tentatively novel species (3). At least 26 of these species, along with many serogroups, are known to cause human infections upon inhalation of aerosolized bacteria (4, 5). *Legionella cardiaca* is a species described in 2012 of a lone strain that was initially isolated in August 2009 in the Chicago area from an aortic valve abscess of a 68-year-old woman with endocarditis, pneumonia, and kidney transplant (6, 7). Since then, no other cases of infection have been reported. It remains to be seen whether *L. cardiaca* may cause Legionnaires’ disease in view of the lack of prior pneumonia workup. Here, we describe two such pneumonia cases in patients with underlying chronic hematologic malignancy.

## CASE PRESENTATION

### Case 1

The first patient was a 73-year-old man and ex-smoker from New Orleans, Louisiana, USA. He was diagnosed with multiple myeloma in 2015; since then, he has endured many antineoplastic treatments, including an autologous stem cell transplant in 2016, all at our institution in Houston. At the end of July 2022, he completed another chemotherapy cycle consisting of carfilzomib, pomalidomide, and dexamethasone.

In mid-August 2022, the patient presented to the emergency department (ED) with night sweats, subjective fever, fatigue, and productive cough for a duration of two days after the start of a new round of chemotherapy in the clinic. His vital signs showed a fever (38.1°C) and a heart rate of 86/min without distress. Laboratory examinations revealed leukocytosis with white blood cell counts of 11,400 × 10^6^/L two days earlier in the clinic and 7,400 × 10^6^/L with left shift in the ED, from his usual levels of 3,500–5,000 × 10^6^/L a few weeks earlier. Mild anemia and thrombocytopenia with hemoglobin of 12.7 g/dl and a platelet count of 106 × 10^9^/L were also found. A chest X-ray film showed left perihilar opacity to further support a working diagnosis of pneumonia. A blood culture was drawn. A nasopharyngeal swab was obtained and tested negative for SARS-Cov-2 virus, influenza A and B viruses, respiratory syncytial virus, adenovirus, *Chlamydia pneumoniae*, and *Mycoplasma pneumoniae*. The patient was treated empirically with intravenous cefepime (2 g q8h) for broad coverage and admitted to the hospital.

**Fig 1:**
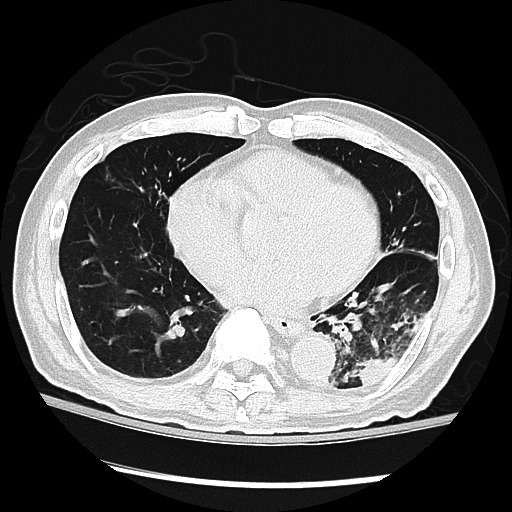
CT of *L. cardiaca* pneumonia in a 73-year-old man showing consolidative and ground-glass opacities in the left lower lobe and ground-glass appearance and bronchiectasis in the other lobes.

A computed tomography (CT) scan on day 2 of hospitalization showed an interval increase in ground-glass and consolidative opacities in the left lower lobe with a small left pleural effusion and new ground-glass opacities in the right upper lobe, suggesting bilateral pneumonia (Fig. 1). Pre-existing tree-in-bud bronchiectasis and ground-glass opacities in the other lobes were also noted. These findings prompted the addition of doxycycline to the antimicrobial treatment. The etiology of the pneumonia was sought further. A blood cytomegalovirus test by polymerase chain reaction (PCR) was positive for 80 IU/mL viral DNA, consistent with low-level viremia due to chronic reactivation noted previously. Urine antigens for *L. pneumophila* serotype 1 and *Streptococcus pneumoniae* were tested negative. A bronchoscopy was performed on day 4, and cloudy lavage fluid from the left lower lobe was sent for PCR tests, cytologic examination, and cultures. The PCR tests revealed 428 IU/mL cytomegalovirus, which was deemed low level and contributive to the pneumonia but unlikely causative. No other respiratory viruses or *Pneumocystis jiroveci* were detected. The cytologic examination revealed rare leukocytes, somewhat surprising but consistent with the use of dexamethasone, a strong and long-acting immune suppressor (8). The blood culture showed no growth. The patient’s condition improved with treatments, and he was discharged after 7 days. The intravenous antibiotics were switched to oral amoxicillin–clavulanate along with ganciclovir to treat cytomegalovirus reactivation.

The bronchoalveolar lavage (BAL) fluid was cultured for bacteria, fungi, and mycobacteria. The culture on buffered charcoal yeast extract (BCYE) agar showed growth of a Gram-negative, slender, and faintly stained bacillus upon 5 days of incubation. The growth was also dependent on L-cysteine. These features pointed to the likelihood of *Legionella*. However, routine profiling by matrix-assisted laser desorption/ionization time-of-flight mass spectrometry yielded no identification. This led to sequence analyses of the 16S rRNA gene and Mip gene. The 16S gene method, as described previously (5, 9, 10), used three sets of primers for PCR amplification, determined the amplicon sequences by the Sanger method, and queried the GenBank database for matches. The Mip method amplified the gene with two M13-tagged primers, 5′-TGTAAAACGACGGCCAGTATGTCTACGGCAATTGTTGC and 5′-CAGGAAACAGCTATGACCCCACCGACGCTACGTGGAC. The results yielded complete matches with the 16S gene (1,448 of 1,448 nucleotides) and the Mip gene (634 of 634) of *L. cardiaca* genome (GenBank accession CP119078.1) (6) to render a confident identification. The next match was *Legionella brunensis* at 98.4% (1,415 of 1,438) for the 16S gene and 85.4% (540 of 632) for the Mip gene. All other cultures, when completed, did not recover significant organisms. Therefore, with the possible contribution of cytomegalovirus, *L. cardiaca* was considered the cause of this patient’s pneumonia.

The patient was followed weekly and monthly after discharge; he had no complaints with health status returned to his baseline. On inquiry, no clear exposure source or infection cluster was noticed, making this case a sporadic infection. However, the exposure should be in New Orleans in early August in view of the usual incubation period of 4–14 days for Legionnaires’ disease and his arrival at Houston by airplane within a day of the onset. This implies the presence of *L. cardiaca* in the New Orleans area, in addition to the Chicago area (6). Weather records in New Orleans showed rainfall in 9 of the 10 days in early August. It is known from earlier studies that sporadic cases of Legionnaires’ disease may follow rainfalls by several days to 2 weeks to approximate the incubation period (11, 12). The biological basis for this rainfall effect, realized recently, is that soil *Legionella* spp. on wet roads during and/or after rainfall may be aerosolized by motor vehicle traffic to expose the drivers, passengers, and possibly pedestrians nearby (13, 14). In this regard, road exposure can be speculated for this patient who had driven in New Orleans in those days.

### Case 2

The second patient was a 67-year-old Caucasian man from Oklahoma City, Oklahoma, USA, with a diagnosis of chronic lymphocytic leukemia. The diagnosis was made in 2012, for which he was treated with fludarabine, cyclophosphamide, and rituximab, due to the involvement of lymph nodes and bone marrow. Since 2015, he has received care for the leukemia at our institution. He is treated currently with acalabrutinib for the leukemia progression. The patient also had a long history of diabetes mellitus with HbA1c around 7.6%.

In mid-July 2024, the patient developed a nonproductive cough and low-grade fever for two days and presented to the clinic for follow-up. A fever of 39.2°C was found, which prompted admission to the hospital. A chest X-ray showed bilateral scattered pulmonary opacities, suspicious for pneumonia. Laboratory examinations revealed pancytopenia, including a white blood cell count of 3,300 × 10^6^/L with 85% neutrophils (relative neutrophilia), lymphopenia (lymphocyte count of 430 × 10^6^/L), anemia (hemoglobin of 9.6 g/dl), and thrombocytopenia (platelet count of 116 × 10^9^/L). A blood culture was drawn. The patient was treated empirically with cefepime (2 g q8h), linezolid (600 mg bid), and azithromycin (500 daily). Notably, the lymphopenia has been long-standing.

The diagnostic workup continued with a negative urine *Legionella* antigen test. The next day, a CT scan showed new bilateral lower lung consolidations, patchy ground-glass opacities, and scattered lung nodules, likely representing pneumonia. A bronchoscopy was performed, and the BAL fluid was tested extensively, such as tests for respiratory viruses, cytomegalovirus, Aspergillus antigen, and *P. jiroveci* that were all negative; a cytologic exam that showed acute inflammation; and cultures for bacteria, fungi, and mycobacteria.

The BAL cultures grew a slender Gram-negative bacillus on the BCYE agar after 4 days of incubation. The growth of the bacterium was also dependent on L-cysteine. However, it was not identified by mass spectrometry. Like the previous case, two genes were analyzed, which matched with *L. cardiaca* in the 16S gene (1,375 of 1,375) and the Mip gene (627 of 627). No other bacteria, fungi, or mycobacteria were isolated. Thus, *L. cardiaca* was deemed to be the cause of pneumonia.

The patient’s pneumonia resolved upon treatment for 10 days, leading to discharge and further treatment with oral levofloxacin. An inquiry on possible exposure was made. The patient had been at home in Oklahoma City within the 3 weeks before the clinic visit, implying local exposure and the presence of *L. cardiaca* there. He had used a humidifier during sleep for mild sleep apnea at home and during hospital stay. The apparatus was cultured, but no *Legionella* or other significant bacteria were recovered. Significantly, 4 days prior to the clinic visit, he performed substantial yard work without wearing a mask at his lake house, including mowing the lawns, raking damp moldy tree leave piles, and operating a mulching machine. The house was within walking distance of a lake, and tree leaves had built up for several months to mimic compost. Therefore, in view of the reported presence of *Legionella* in compost (15), the mulching work was considered the most likely source of bioaerosols for this patient.

## DISCUSSION

Patients with cancer, particularly hematologic malignancies, are prone to various kinds of infections, such as pneumonia due to *L. pneumophila* and many other low pathogenic *Legionella* species (5), systemic cytomegalovirus reactivation (16), bacteremia due to environmental bacteria (10), and other opportunistic infections. In those with *Legionella* pneumonia, consolidation and ground-glass opacities on CT scans are common findings (17). The patients here represent such examples of infection due to a rare *Legionella* species along with cytomegalovirus reactivation in one of them. Several factors predispose them to Legionnaires’ disease, including lymphoid malignancy, antineoplastic treatments, and cytopenia. The use of dexamethasone, history of smoking with bronchiectasis, and older age also contributed to the first case. These cases illustrate the importance of bronchoscopy and culture in pinpointing the etiologic agent of pneumonia when facing negative findings of urine antigen tests and other common pathogens.

The case of *L. cardiaca* infection reported previously shared some features with our patients: pneumonia, immune suppression for the prevention of rejection of renal transplant, episode of earlier cytomegalovirus reactivation, age above 65 years, and the time of infection in August (6). The patient’s endocarditis was a severe complication; presumably, the native aortic valve leaflets were seeded by occult or transient bacteremia that might have developed days to weeks earlier from *L. cardiaca* pneumonia. It is known that concurrent bacteremia in *L. pneumophila* pneumonia may occur (5, 18). Unfortunately, despite valve replacement surgery, the patient succumbed to further complications several months later. Together, the three cases so far suggest that *L. cardiaca* is an opportunistic pathogen to cause pneumonia. In immunocompromised patients, Legionnaires’ disease due to *Legionella* spp. other than *L. pneumophila* may be subacute and lingering in our experience (5).

The origin of *L. cardiaca* is likely environmental, just like other *Legionella* species. In the GenBank, a direct deposit of the Mip gene (JN380975 by RM Ratcliff on 25 July 2016) matched *L. cardiaca* completely and showed isolation of the strain *Legionella* sp. Pru-2 from cooling tower water in the Czech Republic. Our second patient here also had an exposure to compost (or soil) as a possible source.

Legionnaires’ disease, Pontiac fever—a less common and mild *Legionella* infection, and rare extrapulmonary infection are termed legionellosis generically. The legionellosis incidence in the United States has increased rapidly in the past two decades from 1,108 cases in 1999 to 9,933 cases in 2018 with age-adjusted incidence rates of 0.40/100,000 to 2.69/100,000, a 6.8-fold hike (14, 19). The increase has been attributed mainly to climate changes with above-normal temperatures and precipitation surplus (incubator effect) and increasing motor vehicle traffic with road exposure to aerosolized soil bacteria (14). Most cases are sporadic, accounting for 94.8% of all cases (X. Y. Han, unpublished data), and occurring mainly in the warm months of June to October (13). The states east of the Mississippi River show higher incidence rates than the western states do, coinciding with more precipitation (13, 19). Furthermore, the northeastern states bear the highest incidence rates, led by Ohio, New York, Pennsylvania, and Michigan, due to mainly relatively weak solar strength in contrast to strong sunlight in the south that reduces road exposure (13, 14). These recent insights may prove to be useful in the prevention of rising legionellosis incidence.
